# DAZAP1 regulates the splicing of *Crem*, *Crisp2* and *Pot1a* transcripts

**DOI:** 10.1093/nar/gkt746

**Published:** 2013-08-21

**Authors:** Hsiang-Ying Chen, Yueh-Hsiang Yu, Pauline H. Yen

**Affiliations:** ^1^Graduate Institute of Life Sciences, National Defense Medical Center, Taipei 11490, Taiwan and ^2^Institute of Biomedical Sciences, Academia Sinica, Taipei 11529, Taiwan

## Abstract

Deleted in Azoospermia Associated Protein 1 (DAZAP1) is a ubiquitous heterogeneous nuclear ribonucleoprotein (hnRNP) that is expressed abundantly in the testis. DAZAP1 deficiency in mice results in growth retardation and spermatogenic arrest. Previous reports on DAZAP1’s binding to several naturally occurring splicing mutations support a role for DAZAP1 in RNA splicing. To elucidate the biological function(s) of DAZAP1 and to search for its natural RNA substrates, we used microarrays to compare the expression profiles and exon usages of wild-type and *Dazap1* mutant testes and identified three genes (*Crem*, *Crisp2* and *Pot1a*) with aberrant RNA splicing in the mutant testes. We further demonstrated that DAZAP1, but not DAZAP1 mutant proteins, promoted the inclusion of *Crem* exon 4, *Crisp2* exon 9 and *Pot1a* exon 4 in splicing reporter transcripts in cultured cells. Additional studies on the binding of DAZAP1 to the exons and their flanking intronic sequences and the effects of minigene deletions on exon inclusion identified regulatory regions in *Crem* intron 3, *Crisp2* intron 9 and *Pot1a* intron 4 where DAZAP1 bound and regulated splicing. Aberrant splicing of the *Pot1a* gene, which encodes an essential protein that protects telomere integrity, may partially account for the growth retardation phenotype of DAZAP1-deficient mice.

## INTRODUCTION

Most eukaryotic genes contain introns that are spliced out soon after they are transcribed. RNA splicing is catalysed by the spliceosome, which consists of five small nuclear ribonucleoprotein particles (snRNPs) and a host of RNA-binding proteins that participate in and regulate the event (1). In addition to the 5′ and 3′ splice sites, the branch point and the polypyrimidine tract, which are recognized by the basic components of the spliceosome, RNA splicing also involves many other cis-acting regulatory elements. Based on the location and the effect, these elements are categorized as exonic splicing enhancers (ESEs), exonic splicing silencers (ESSs), intronic splicing enhancers (ISEs) and intronic splicing silencers (ISSs). They are recognized by trans-acting RNA-binding proteins that regulate either or both constitutive and alternative splicing events to determine the inclusion/exclusion of a particular exon. There are two major families of RNA-binding proteins that regulate RNA splicing. The SR (Ser-Arg) proteins in the first family are characterized by one or two N-terminal RNA recognition motif (RRM) domains and a C-terminal RS domain enriched in Arg-Ser dipeptides (2). Most SR proteins bind to ESEs and activate both constitutive and alternative splicing by facilitating splice site recognition (3). Some of these SR proteins are involved in additional cellular functions such as mRNA export, stability and translation. The second family consists of heterogeneous nuclear ribonucleoproteins (hnRNPs) (4). Over half of the 20 or so known hnRNP proteins have been shown to participate in pre-mRNA splicing (1). Most of them, including hnRNP A1, negatively regulate splicing by binding to ESSs and ISSs to block the access of snRNPs to splicing signals or positive regulatory factors to enhancers. Some hnRNP proteins, such as hnRNP F and hnRNP H, bind ISEs instead to stimulate splicing (5). An increasing number of proteins have been reported to function both as repressors and activators depending on the position of their binding site related to the exons that they regulate (6). Thus, RNA splicing is regulated by a host of RNA-binding proteins, some of which are expressed constitutively in all cells whereas others are expressed in tissue or developmental specific manners. Such differential expression of splicing regulatory factors is responsible for the alternative RNA splicing that has greatly increased the complexity of the transcriptome.

Deleted in Azoospermia Associated Protein 1 (DAZAP1) is a more recently identified hnRNP protein that shares several characteristics with hnRNP A1, such as containing two N-terminal RRMs, ubiquitous expression, nucleocytoplasmic shuttling and transcription-dependent nuclear localization (7–9). DAZAP1 interacts with several RNA-binding proteins, including DAZ, DAZL, hnRNPA1, hnRNP A/B, hnRNP D, hnRNP U like 1 and DDX20 (7,10). It also contains a prion-like domain that makes it prone to form toxic cytoplasmic aggregates when over-expressed in yeast (11). DAZAP1 is expressed most abundantly in the testis, present predominantly in the nuclei of late-stage spermatocytes and round spermatids, and the cytoplasm of elongated spermatids (12). It is also present in subnuclear paraspeckles that contain long non-coding RNAs (13). DAZAP1 has been implicated in several cellular processes, including transcription (14,15), mRNA translation (16) and RNA splicing (17–19). The evidence supporting a role for DAZAP1 in RNA splicing so far came from studies on pathological splicing mutations. The first two cases involve the *Neurofibromatosis* (*NF1*) and *Breast Cancer 1* (*BRCA1*) genes where mutations within the exons created new ESSs that were recognized by hnRNP A1/A2 as well as DAZAP1 and resulted in exon skipping (17,18). In a third case, an Alu-derived ISE in the *Ataxia Teleangectasia Mutated* (*ATM*) gene caused the inclusion of a cryptic exon, and hnRNPA1 and DAZAP1 were found to act negatively and positively, respectively, on the exon selection (19). In these three cases, the sequences to which DAZAP1 bound were created by mutations. Thus, the natural substrates of DAZAP1 remain elusive.

We recently generated two *Dazap1* mutant alleles in an attempt to elucidate the biological function(s) of DAZAP1 (20). The *null* allele produced no DAZAP1 protein, whereas the *Floxed with Neo* (*Fn*) allele made an aberrant DAZAP1-Fn protein, which had an insertion of a 166 amino acid segment between the two RRMs. Most mice homozygous for either the *null* or the *Fn* allele died perinatally. The few survivors could live to adulthood but manifested growth retardation and spermatogenic failure. Male adult mutant mice had small testes that contained fully developed spermatocytes but no post-meiotic germ cells. The phenotype of *Dazap1* mutant mice indicates that DAZAP1 is essential for normal mouse development and spermatogenesis. The availability of *Dazap1* mutant mice enabled us to search for DAZAP1 down-stream targets using microarray approaches. Here, we report our identification of *Crem*, *Crisp2* and *Pot1a* as the natural substrates of DAZAP1 and show that DAZAP1 promotes the inclusion of specific exons in these genes.

## MATERIALS AND METHODS

### Animals

Mice used in the study were of a mixed genetic background between C57BL/6 and 129 strains. They were housed in a specific pathogen-free animal facility, and the experiments were approved by the Institutional Animal Care & Utilization Committee of Academia Sinica. Mice homozygous for the *Dazap1 floxed with neo* (*Fn*) allele were generated by crossing between the *Dazap1^+/Fn^* heterozygotes as previously described (20). Adult mice were 2 ∼ 6 months old.

### Microarray processing and data analysis

Total RNAs were extracted from mouse testes with Trizol (Invitrogen, Carlsbad, CA). Microarrays were processed by the Affymetrix Gene Expression Service Lab (AGESL) in Academia Sinica (Taipei, Taiwan), and three independent experiments were performed using different RNA preparations. The Affymetrix Mouse Genome 430 2.0 GeneChip (Affymetrix Inc., Santa Clara, CA) data were analysed using the GeneSpring 7.3 Expression Analysis Software (Agilent Technologies, Santa Clara, CA), whereas the Affymetrix-GeneChip Mouse Exon 1.0 ST array data were analysed with the EASANA analysis system using the service of GenoSplice Technology (Paris, France).

### RT-PCR

Total RNAs were reverse-transcribed (RT) into cDNA by SuperScript III reverse transcriptase using Oligo(dT)_20_ and random hexamers as primers according to the manufacturer’s instruction (Invitrogen). One tenth of the RT products were used as templates together with specific primers (Supplementary Table S1) in subsequent PCR reactions. PCR conditions are given underneath the supplementary table. The products were separated on 2% agarose gels, stained with EtBr, and photographed with the Quantum ST4 imaging system (Vilber Lourmat, Eberhardzell, Germany). The signals were quantified using the Gel-Pro Analyzer program (Media Cybernetics, Warrendale, PA), and statistical analyses were carried out by use of the GraphPad Prism software (GraphPad Software, La Jolla, CA).

### Western blots

Tissue culture cells or mouse testes were lysed/homogenized in the RIPA buffer (50 mM Tris pH 8.0, 150 mM NaCl, 0.1% SDS, 0.5% deoxycholate, 1% NP-40), and protein concentrations were determined by Bradford assays (Bio-Rad, Hercules, CA). About 40 µg of total protein was electrophoresed on 10% SDS-PAGE gels and transferred to PVDF membranes (Merck Millipore, Billerica, MA). The blots were probed with antibodies against CREM (sc-440, Santa Cruz Biotechnology Inc, Santa Cruz, CA), CRISP2 (19066-1-AP, Proteintech, Chicago, IL), POT1A (21), DAZAP1 (22) or β-ACTIN (A5441, Sigma, Saint Louis, MI). The signals were detected using an enhanced ECL chemoluminescence system (Merck Millipore) followed by the BioSpectrum Multispectral Imaging System (UVP, Upland, CA).

### Cell culture and transfection

Mouse embryonic fibroblasts (MEFs) were prepared from E14.5 embryos (23). MEFs and COS7 cells were maintained in DMEM (Hyclone, Fremont, CA) supplemented with 10% fetal bovine serum and 1× penicillin/streptomycin (GIBCO, Eggenstein, Germany). The cultures were incubated at 37°C in a humidified 5% CO_2_ atmosphere. When the cells were grown to 50 ∼ 70% confluency, they were transfected with plasmid DNAs using the TurboFect reagent (Fermentas, Burlington, Ontario, Canada) for COS7 cells and the PolyJet reagent (SignaGen Laboratories, Gaithersburg, MD) for MEFs.

### Minigene construction and splicing analysis

Fragments for minigene constructs were PCR amplified from mouse genomic DNA using primers containing specific restriction sites (Supplementary Table S2) and cloned into the pcDNA3.1(+) expression vector (Invitrogen). Minigenes with specific deletions as well as the expression vector for DAZAP1-ΔRRM were generated by a PCR-mediated deletion approach (24). For the splicing analysis, 0.5 µg of the minigene was co-transfected with 1.5 µg of the appropriate expression vector into COS7 or MEF cells in a 6-cm dish. In the experiments comparing the effects of DAZAP1-Wt, DAZAP1-Fn and DAZAP1-ΔRRM on exon inclusion (Figure 3B), 0.375 µg (instead of 1.5 µg) of the DAZAP1-Wt expression vector was used. The cells were harvested 24 hours later, and total RNA was isolated and reverse transcribed. PCR amplification was carried out using a forward primer specific to the upstream exon (Supplementary Table S2) and a reverse primer BGH-R (5′-tagaaggcacagtcgagg) containing the vector sequence downstream of the cloning site. The products were separated on agarose gels, and the signals of the DNA fragments were analysed by the Gel-ProAnalyzer and GraphPad Prism programs. Percentage of splicing inclusion (PSI) (25) was calculated as the signal of the fragment including the exon divided by the total signals of the fragments with and without the exon.

### Protein expression and purification

The coding sequence of *DAZAP1* cDNA was cloned into the pGEX-5X-1 expression vector (GE Healthcare, Piscataway, NJ) and then expressed in BL21 (DE3) under the induction of 1 mM IPTG at 20°C for 4 h. The recombinant GST-DAZAP1 fusion protein was purified with glutathione S-Sepharose 4B beads according to the manufacturer’s instruction (GE Healthcare).

### Electrophoretic mobility shift assay

To make RNA probes, exon/intron fragments of *Crem* were cloned into the pBluescript vector (Stratagene, La Jolla, MA, USA), and those of *Crisp2* and *Pot1a* were cloned into pCRII-TOPO (Invitrogene). The plasmid DNAs were linealized and transcribed into RNAs in the presence of ^32^P-CTP using Riboprobe in vitro Transcription Systems-T7 according to the manufacturer’s instruction (Promega, San Luis Obispo, CA). The resultant RNA transcripts were purified on G-50 mini columns (Geneaid Biotech, Taipei, Taiwan). Electrophoretic mobility shift assay (EMSA) was carried out according to a modified protocol (26). About 10^4^ cpm of ^32^P-labelled RNA probe was incubated without or with 500 nM GST or GST-DAZAP1 in a 20 µl binding reaction containing 20 mM HEPES (pH 7.6), 3 mM MgCl_2_, 40 mM KCl, 2 mM DTT, 5% glycerol and 5 mg/ml heparin. After incubation at room temperature for 15 min, a portion (10 µl) of each sample was separated on native 5% polyacrylamide gels in 0.25× TBE buffer. Gels were dried and visualized with a Typhoon 9410 Imager (GE Healthcare).

To determine the binding affinity between DAZAP1 and its RNA target, about 10^4^ cpm of ^32^P-labelled RNA probe (at a final concentration of 0.5 ∼ 3.0 nM) was incubated with various concentrations (0–500 nM) of GST-DAZAP1. After gel electrophoresis, signals of the shifted species and the total signals in a lane were quantified using the Gel-ProAnalyzer program. The fraction of RNA that bound to DAZAP1 and the GST-DAZAP1 concentration were plotted on the y- and x-axes, respectively, using the Excel software (Microsoft, Redmond, WA). Kd was determined as the protein concentration at which 50% of the RNA was bound (27). Statistical analyses were carried out by use of the GraphPad Prism software.

## RESULTS

### Identification of genes with aberrant splicing in *Dazap1* mutant mice

**Figure 1.**
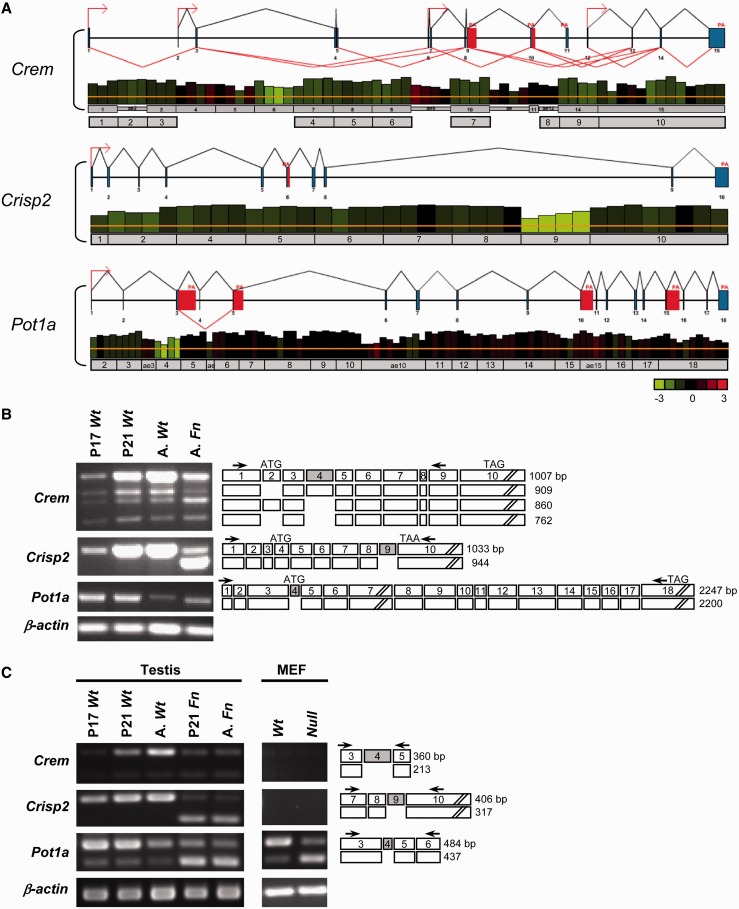
Aberrant splicing of *Crem*, *Crisp2* and *Pot1a* in *Dazap1^Fn/Fn^* mutant mice. (**A**) EASANA analyses of exon arrays of wild type and mutant testis RNAs. For each gene, the upper panel shows the genomic structure, different promoter usage (indicated by red arrows), alternative splicing (indicated by red lines) and alternative polyadenylation (PA) with alternative last exons (indicated with red blocks). In the lower panel, the height of each column represents the mean relative intensity of the probe in three independent experiments for the wild-type testes. The colour of each column indicates differences in probe intensities between wild-type and mutant samples, with red and green representing 3-fold increase and decrease, respectively, in the mutant testes, as indicated at the bottom right corner. The exons of each gene are shown as grey horizontal bars. For the *Crem* gene, the EASANA analyses numbered the exons differently than that indicated in the NCBI *Crem* cDNA sequence NM_001110859. The NCBI exon numbers are shown underneath the EASANA’s numbers and were used in the study. (**B**) RT-PCR of near full-length transcripts in the testes of postnatal day 17 (P17), day 21 (P21) and adult (A) wild type (*Wt*) and *Fn* mutant mice. The exons present in each of the PCR fragments are indicated on the right side. The aberrantly spliced exons are shaded, and the locations of PCR primers are indicated with arrows. The locations of the initiation (ATG) and termination (TAG/TAA) codons are marked, and the sizes of the PCR products are given at right. (**C**) RT-PCR amplification of the aberrantly spliced exons and their flanking exons in *Wt* and *Fn* mutant testes and MEFs established from *Dazap1 Wt* and *null* mutant mice. Percentages of splicing inclusion (PSIs) of the various exons are presented in Supplementary Figure S1.

We initially used the Affymetrix Mouse Genome 430 2.0 array to compare gene expression in the testes of wild-type (*Wt*) and *Dazap1^Fn/Fn^* (*Fn*) mutant mice at day-21 post partum (P21) when both *Wt* and *Fn* testes contained mainly pachytene spermatocytes and few or no haploid spermatids (20). Approximately 2000 and 260 genes showed >2-fold decreases and increases, respectively, in expression level in the mutant testes. Among the down-regulated genes, 87 had previously been implicated in reproduction. We selected 13 genes (*Ccna1, Creb3l4, Crem, Gsg2, Nlrp14, Ovol1, Piwil1, Pxt1, Psme4, Spata16, Tdrd7, Tesk1, Tssk2*) that are expressed abundantly in the testis for further study and confirmed their decreased expression in the mutant testes by real-time RT-PCR (data not shown). However, further investigation failed to detect significant differences between *Wt* and *Fn* testes in both transcriptional rate and mRNA export of these genes, as determined by RNAPol-ChIP (28) and RT-PCR of the nuclear and cytoplasmic transcripts, respectively (data not shown). Additional RT-PCR amplification detected aberrant splicing only in the *Crem* gene (Figure 1). We therefore turned to Affymetrix-GeneChip Mouse Exon 1.0 ST arrays to compare exon usage in *Wt* and *Fn* testes. Concerned that the pachytene spermatocytes in P21 *Fn* testes might be at an earlier stage of development than those in P21 *Wt* testes because of growth retardation of the mutant mice, we switched to use adult *Fn* testes and compared them with P21 *Wt* testes. Adult *Fn* testes contain spermatogonia and spermatocytes at various stages of development, but no haploid spermatids because of spermatogenesis arrest right before meiosis (20). Their germ cell constitution is therefore most similar to P21 *Wt* testes in which close to half of the germ cell population have reached the pachytene stage and a small fraction (∼4%) of them have even completed meiosis (29). The exon array results showed that 1706 and 918 genes had >1.5-fold changes in expression at the gene and the exon levels, respectively, with 418 genes being regulated at both levels. We picked 12 genes with the most significant reduction in the inclusion of one of their exons for RT-PCR study, and confirmed aberrant splicing in the *Fn* testes for only two genes, *Crisp2* and *Pot1a* (Figure 1). The microarray data for both gene expression and exon usage have been deposited in GEO (http://www.ncbi.nlm.nih.gov/geo) under the accession number GSE42604.

### Characterization of aberrantly spliced mRNAs and their protein products

**Figure 2.**
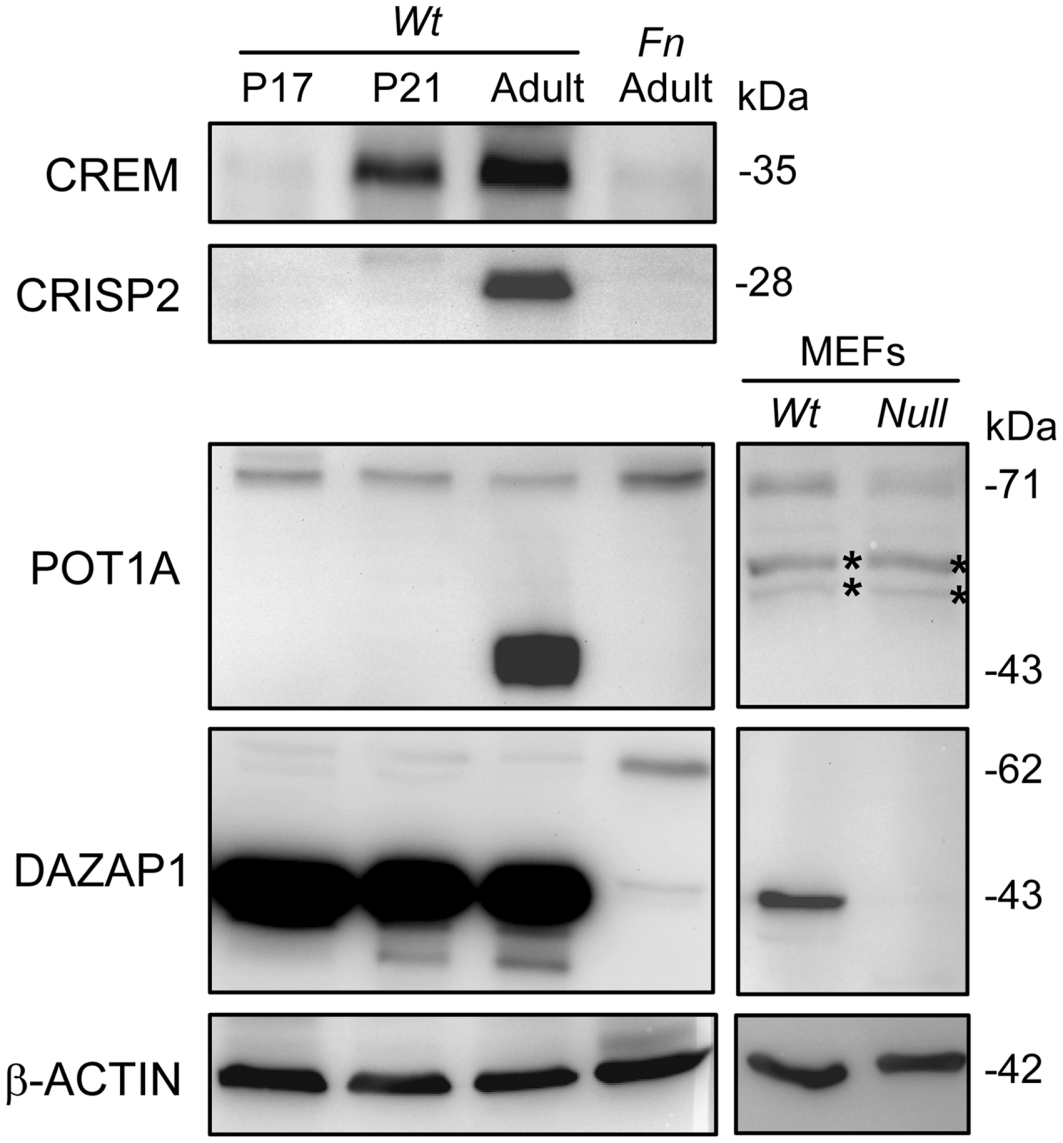
Expression of the protein products of *Dazap1* target genes. Testis lysates of *Wt* mice at the indicated ages and adult *Fn* mutant mice and lysates of MEFs established from *Dazap1 Wt* and *null* mice were western blotted with antibodies against the respective proteins. The non-specific proteins detected by the anti-POT1A antibody are marked with asterisks. The *Fn* mutant allele produced a larger 62 kDa DAZAP1-Fn protein.

*Cyclic AMP responsive element modulator* (*Crem*) encodes a master regulator of several post-meiotic genes, including *Tp1*, *Rt7*, *Cyps1* and Calspermin (30). It has multiple alternatively spliced isoforms producing either active transcription factors or antagonists (31,32). *Crem-*knockout mice manifested spermatogenic arrest at the first step of spermiogenesis (33,34). We studied *Crem* transcripts in prepuberal and adult mouse testes. Postnatal day 17 (P17) and P21 mouse testes contain pachytene spermatocytes and round spermatids as the most advanced germ cells, respectively, and adult testes contain germ cells at all stages of development (29). Our RT-PCR analysis of mouse testis RNA using primers from *Crem* exons 1 and 9 revealed the presence of four *Crem* transcripts, with the longest one being the predominant form (Figure 1B). DNA sequencing of the PCR products indicated that the longest product contained exons 1–9 present in the *Crem* transcript variant 1 (GenBank NM_001110859), whereas the three smaller transcripts lacked exon 2, exon 4 and both exon 2 and exon 4, respectively. In the *Wt* testes, the levels of *Crem* transcripts increased significantly after P17 and reached maximum in the adult, indicating that *Crem* is predominantly expressed in post-meiotic haploid germ cells (Figure 1B). Compared with P21 and adult *Wt* testes, adult *Fn* testes had a much lower total level of *Crem* transcripts. However, the relative levels of the two *Crem* transcripts lacking exon 4 were significantly increased. Additional RT-PCR analyses across exon 2 alone or exon 4 alone showed the increase in transcripts lacking exon 4 (Figure 1C), but not exon 2 (data not shown), in both P21 and adult *Fn* testes. The percentage of splicing inclusion (PSI) of exon 4 decreased from 89.8 ± 8.0% in the P21 *Wt* testes to 67.1 ± 0.9% (*P* = 0.046) and 63.8 ± 1.5% (*P* = 0.024) in P21 and adult *Fn* testes, respectively (Supplementary Figure S1A). We would not have picked up *Crem* as having aberrant splicing in the *Fn* testes by the exon arrays since the under-representation of its exon 4 in the *Fn* testes was not obvious (Figure 1A). *Crem* exon 4 encodes a glutamine-rich trans-activation domain, and its deletion does not cause reading frame shift. Western blotting of mouse testis lysates detected only the 35 kDa CREM protein, translated from the most abundant *Crem* transcript containing all exons (Figure 2). The level of CREM in the *Wt* testes increased as the mice grew into adulthood whereas that in the adult *Fn* testes remained low, showing a general agreement with the *Crem* transcript levels in the various testes.

Cysteine-rich secretory protein 2 (CRISP2), also known as testis-specific protein 1 (TPX-1), is a testis-enriched protein that is present in the acrosome and the tail of sperm and has been implicated in sperm-oocyte binding and sperm mobility (35,36). Our exon array results showed that *Crisp 2* exon 9 was under-represented in the *Fn* testes (Figure 1A), which was further confirmed by RT-PCR across all *Crisp 2* exons (Figure 1B) or just a few flanking exons (Figure 1C). In *Wt* testes, the level of the *Crisp2* transcript was low at P17 and increased significantly at P21, indicating again that *Crisp2* is expressed predominantly in post-meiotic germ cells (Figure 1B). P21 and adult *Fn* testes contained a significantly reduced level of the full-length *Crisp2* transcript and a high level of a new transcript lacking exon 9. The PSI of exon 9 decreased from 100% in both P21 and adult *Wt* testes to 16.6 ± 2.0% (*P* = 0.0002) and 14.6 ± 0.8% (*P* < 0.001), respectively, in P21 and adult *Fn* testes (Supplementary Figure S1A). *Crisp2* exon 9 is 89 bp long, and its skipping would change the reading frame of the last exon 10 and replace the C-terminal 71 amino acid residues of the 243 amino acid long protein with a single amino acid residue. Because the premature stop codon was still in the last exon, the *Crisp2* transcript lacking exon 9 might not be a target of nonsense–mediated decay (37), as suggested by the high level of its presence in the *Fn* testes. Western blotting on testis lysates showed the presence of CRISP2 only in adult *Wt* testes (Figure 2). The absence of CRISP2 protein products in P21 *Wt* testes and adult *Fn* testes despite high levels of *Crisp2* transcripts suggests mRNA translation repression, which has been reported for several post-meiotically expressed genes during spermatogenesis (38).

Protection of telomeres 1a (POT1A) is a component of the shelterin complex that is present at the ends of mammalian chromosomes (39). It binds to the 3′ single-stranded G-rich overhangs at the telomeres and plays an essential role in protecting the chromosomal end and regulating telomere length. *Pot1a* KO mice die during early embryonic development (21,40). Our exon array data picked up *Pot1a* exon 4 as under-represented in the *Fn* testes (Figure 1A). This was confirmed by RT-PCR across exons 3–6 (Figure 1C). In *Wt* testes, the levels of *Pot1a* transcripts were similar at P17 and P21 but consistently lower in the adult. Nonetheless, the PSI for exon 4 remained unchanged at around 79%. Both P21 and adult *Fn* testes contained a comparable total level of *Pot1a* transcripts, but only 35.5 ± 0.6% (*P* < 0.0001) and 30.6±1.3% (*P* = 0.0005), respectively, of the transcripts included exon 4 (Supplementary Figure S1A). MEFs also expressed high levels of *Pot1a*, but not *Crem* or *Crisp2* (Figure 1C). As in the testes, the vast majority (PSI = 80.0 ± 1.3%) of *Pot1a* transcripts in *Wt* MEFs included exon 4, whereas only a quarter of *Pot1a* transcripts (PSI = 25.2 ± 3.7, *P* = 0.0012) in the *null* MEFs contained exon 4 (Figure 1C and Supplementary Figure S1B). *Pot1a* exon 4 contains the translation initiation codon, and a *Pot1a* transcript without exon 4 is predicted to be translated from a downstream AUG codon to produce a shorter 64 kDa protein lacking the first 59 amino acid residues. We examined the protein products of *Pot1a* transcripts by western blotting using an antibody raised against POT1A amino acids 395–421 (21). The testes of *Wt* mice at different ages gave a band of comparable intensity at 71 kDa, the size of the full-length POT1A (Figure 2). In the adult testes there was an additional very intense band at 43 kDa that could represent the product of an alternatively spliced transcript not detected by our PCR reactions, or an unrelated cross-reacting protein. Several splice variants of human *POT1* transcripts have been reported (41), but mouse *Pot1a* splice variants have yet to be characterized. Adult *Fn* testes also showed a single band at 71 kDa that was unexpectedly more intense than those in the *Wt* testes, and there was no sign of a truncated 64 kDa protein. The inconsistency between the levels of *Pot1a* transcripts and POT1A proteins in the *Fn* testes suggests that the intensity of the band at 71 kDa might be partially contributed by a cross-reacting protein and that the predicted 64 kDa protein is likely unstable. Additional western blotting on MEFs that had been established from *Dazap1 null* mice and their *Wt* littermates (20) showed a significantly reduced level (42 ± 6%, *P* = 0.0039) of the 71 kDa protein in the *null* MEFs as compared with the *Wt* MEFs (Figure 2 and Supplementary Figure S2), in agreement with the RT-PCR results.

In conclusion, our analyses of the alternatively/aberrantly spliced transcripts of *Crem*, *Crisp2* and *Pot1a* in *Fn* mutant mice showed that DAZAP1 deficiency resulted in partial exclusion of a specific exon in each of these target genes.

### Enhancement of exon inclusion in minigenes by DAZAP1

**Figure 3.**
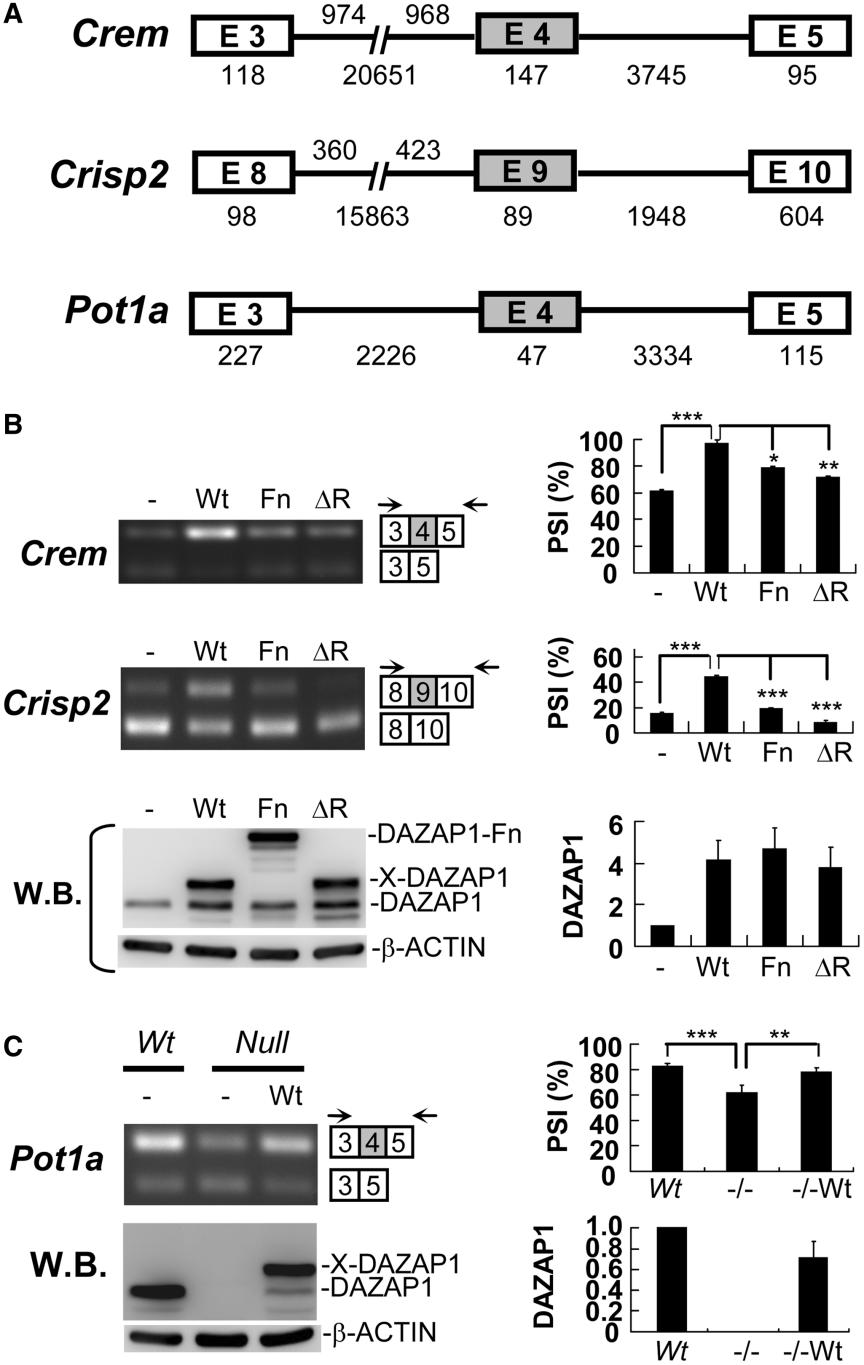
Enhancement of exon inclusion by DAZAP1. (**A**) Structures of the splicing reporter minigenes. The boxes and lines represent exons and introns, respectively, with the testing exons shown in grey and the sizes (in base pairs) of the exons and intact introns given underneath. The *Crem* and *Crisp2* minigenes contain segments of the upstream introns and the sizes of the segments are indicated above the introns. (**B**) Minigene splicing assays in COS7 cells. COS7 cells were transfected with the *Crem* or *Crisp2* minigenes together with the empty Xpress cloning vector (−), or the expression vector for Xpress-tagged wild-type DAZAP1(Wt), DAZAP1-Fn mutant (Fn) or DAZAP1-ΔRRM (ΔR) as indicated. The expression of the minigenes was detected by RT-PCR using gene-specific and common primers, indicated as arrows. Bar charts at right show the PSI for the targeted exons. The expression of the various DAZAP1 proteins in the transfected cells was detected by western blotting (WB) using an anti-DAZAP1 antibody which detected the endogenous DAZAP1, Xpress-tagged DAZAP1-Wt and -ΔR proteins (X-DAZAP1), and the DAZAP1-Fn mutant. The relative levels of total DAZAP1 proteins in the cells, shown at right, were determined using that in the cells transfected with the empty vector as a reference. (**C**) Minigene splicing assays in MEFs. MEFs established from *Dazap1 Wt* and *null* mutants were transfected with the *Pot1a* minigene together with the empty-vector or DAZAP1-Wt expression vector, and the minigene expression was detected by RT-PCR. Western blotting of DAZAP1 in the cells lysates is shown at bottom. Quantitative analyses of the signals are shown at right. The results shown represent the average of three independent experiments. Statistical significance of the difference was determined using the paired t test. * denotes *P* < 0.05, ** denotes *P* < 0.01 and *** denotes *P* < 0.001.

We next constructed splicing reporter minigenes containing the under-represented exons identified above and portions of their flanking introns and exons (Figure 3A), and transfected these minigenes into cultured cells to study the effects of exogenous Xpress-tagged DAZAP1 on exon inclusion (Figure 3B and Supplementary Figure S3). We included two mutants, DAZAP1-Fn and DAZAP1-ΔRRM, in addition to the wild-type DAZAP1-Wt protein in the study. DAZAP1-Fn, with a large insertion between the two RRMs, might have retained some residual activity. This was suggested by the observation that more *Fn* mutants than *null* mutants survived the first week of life and lived to adulthood (20). DAZAP1-ΔRRM had lost its RNA binding ability due to the replacement of four conservative phenylalanine residues with alanine in the two RRM domains (42). We adjusted the quantities of the expression vectors so that similar levels of the various DAZAP1 proteins, as determined by western blotting, were expressed in the transfected cells. In COS7 cells transfected with the minigenes together with the empty expression vector, the PSIs differed for the three minigenes. The two *Crem* transcripts with and without exon 4 were present in comparable levels (PSI = 60.7 ± 1.8%), whereas most *Crisp2* transcripts lacked exon 9 (PSI = 15.6 ± 1.0%) (Figure 3B). Exogenous expression of DAZAP1-Wt significantly increased the inclusion of both *Crem* exon 4 (PSI = 96.7 ± 2.8%, *P* = 0.0008) and *Crisp2* exon 9 (PSI = 44.7 ± 0.7%, *P* = 0.0002). Compared with DAZAP1-Wt, ectopically expressed DAZAP1-Fn and DAZAP1-ΔRRM at similar protein level had significantly less effects on the inclusion of *Crem* exon 4 (PSI = 78.8 ± 0.4%, *P* = 0.01 and PSI = 72.3 ± 1.6%, *P* = 0.0018, respectively) and *Crisp2* exon 9 (PSI = 19.4 ± 0.5%, *P* < 0.0001 and PSI = 8.22 ± 1.7%, *P* = 0.0007, respectively). In COS7 cells, most *Pot1a* reporter transcripts already contained exon 4, making it difficult to study the effect of exogenous DAZAP1 on exon 4 inclusion (Supplementary Figure S3A). We therefore performed additional minigene experiments in MEFs. Unexpectedly, *Wt* and *null* MEFs transfected with the *Crem* or the *Crisp2* minigene showed similar exon inclusion (Supplementary Figure S3B). However, most *Pot1a* reporter transcripts in *Dazap1 Wt* MEF cells contained exon 4 (PSI = 82.5 ± 2.0%), whereas a large fraction of *Pot1a* reporter transcripts in *Dazap1 null* MEF cells lacked exon 4 (PSI = 61.8 ± 6.0%) (Figure 3C). Ectopically expressing DAZAP1-Wt in *Dazap1 null* MEF cells significantly increased the inclusion of exon 4 (PSI = 78.0 ± 3.4%, *P* = 0.0092). The extremely low expression of DAZAP1-Fn and DAZAP1-ΔRRM in transfected *Dazap1 null* MEF cells prohibited us from studying the effects of the DAZAP1 mutants on *Pot1a* exon 4 inclusion.

### Mapping regulatory regions in the target transcripts

**Figure 4.**
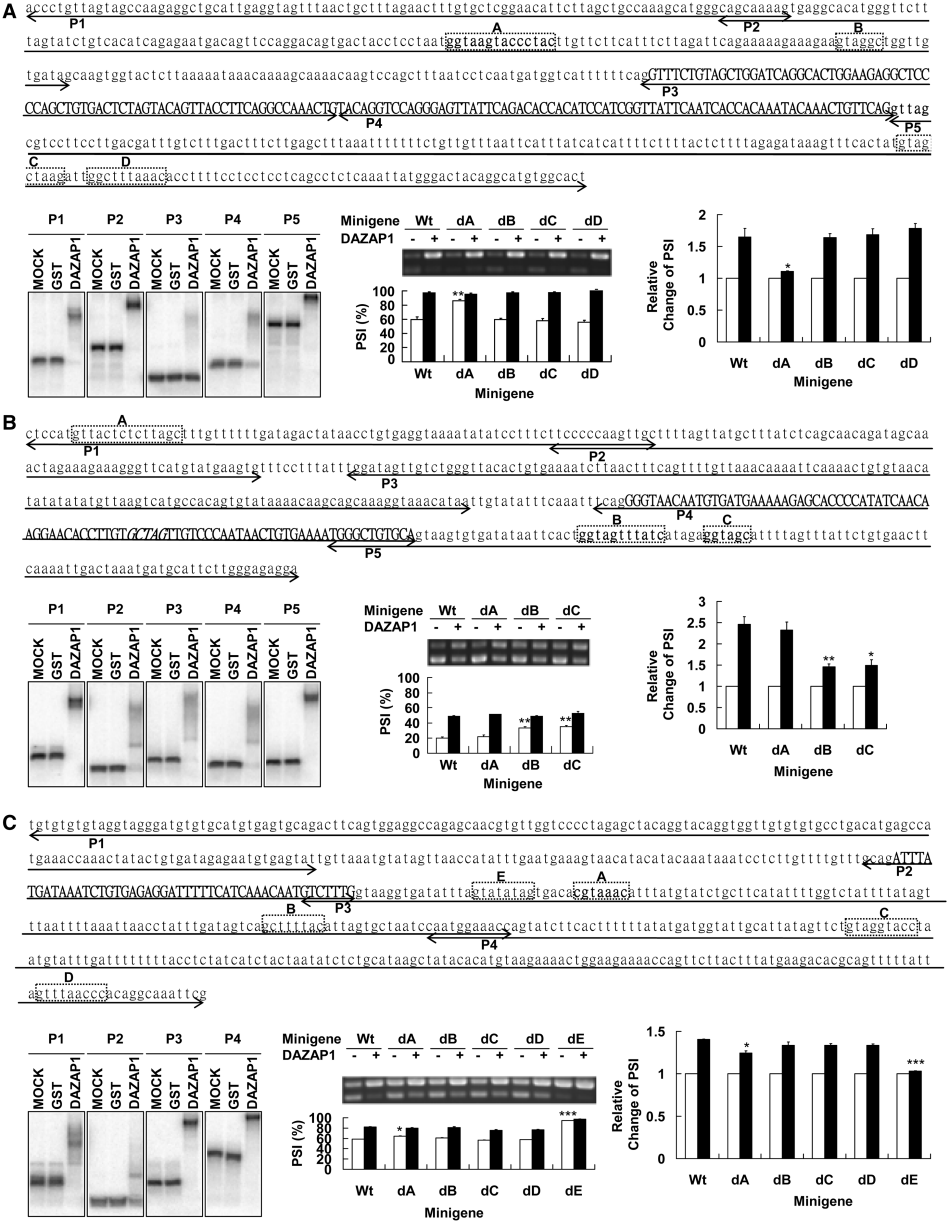
Mapping DAZAP1 binding sites and splicing regulatory regions in the target genes. (**A**) *Crem*, (**B**) *Crisp2* and (**C**) *Pot1a*. For each section dedicated to a specific gene, the sequence of the regulated exon (in Capital bold letters) and its flanking intronic regions (in low case) is shown at top. EMSA probes (P1–P5) are indicated with underlying double-headed arrows, and segments deleted in the various minigene deletion constructs (A–**E**) are boxed. The left panel below the sequence shows the EMSA gel patterns. ^32^P-labelled probes, indicated at top, were incubated with buffer only (mock), GST, or GST-DAZAP1 and analysed by 5% native PAGE. The middle panel shows RT-PCR gel patterns of splicing assays of the various minigene deletion constructs. The PSI of each reaction is shown below the gel lane. The right panel shows the relative change of PSI by DAZAP1, calculated from the middle panel by dividing the PSI value in the presence of exogenous DAZAP1 with that in the absence of exogenous DAZAP1. Three independent experiments were performed, and statistical significance of the differences was calculated using the paired t test. *P < 0.05, **P < 0.01, ***P < 0.001.

Previous studies have identified several DAZAP1-binding sequences. An early SELEX experiment found that most DAZAP1 RNA ligands contained both AAAUAG and GU_1-3_AG sequences (26). More recent characterization of *BRCA1* and *NF1* splice mutations showed that DAZAP1 binds to GGUUAG, GCUUAG and ACUUAG (17,18), and GUAACG was added recently as another sequence recognized by DAZAP1 (43). However, the segment in the *ATM* splice mutant that bound DAZAP1 does not contain any of the above consensus sequences (19). We therefore performed RNA EMSAs followed by splicing assays with truncated minigenes to define the region(s) in each of the target genes that DAZAP1 might bind to regulate splicing (Figure 4). In the EMSAs, we mixed GST-DAZAP1 fusion protein with 4 ∼ 5 RNA probes ( ∼ 70–200 bases in length) corresponding to different areas of the regulated exon and its adjacent intronic sequences and detected the binding by PAGE on native gels. We next selected two regions with the strongest DAZAP1 binding affinity and deleted putative DAZAP1 binding sequences in each region in the minigene construct to study the effects of the deletions on exon inclusion in either COS7 (for *Crem* and *Crisp2*) or MEF (for *Pot1a*) cells.

**Figure 5.**
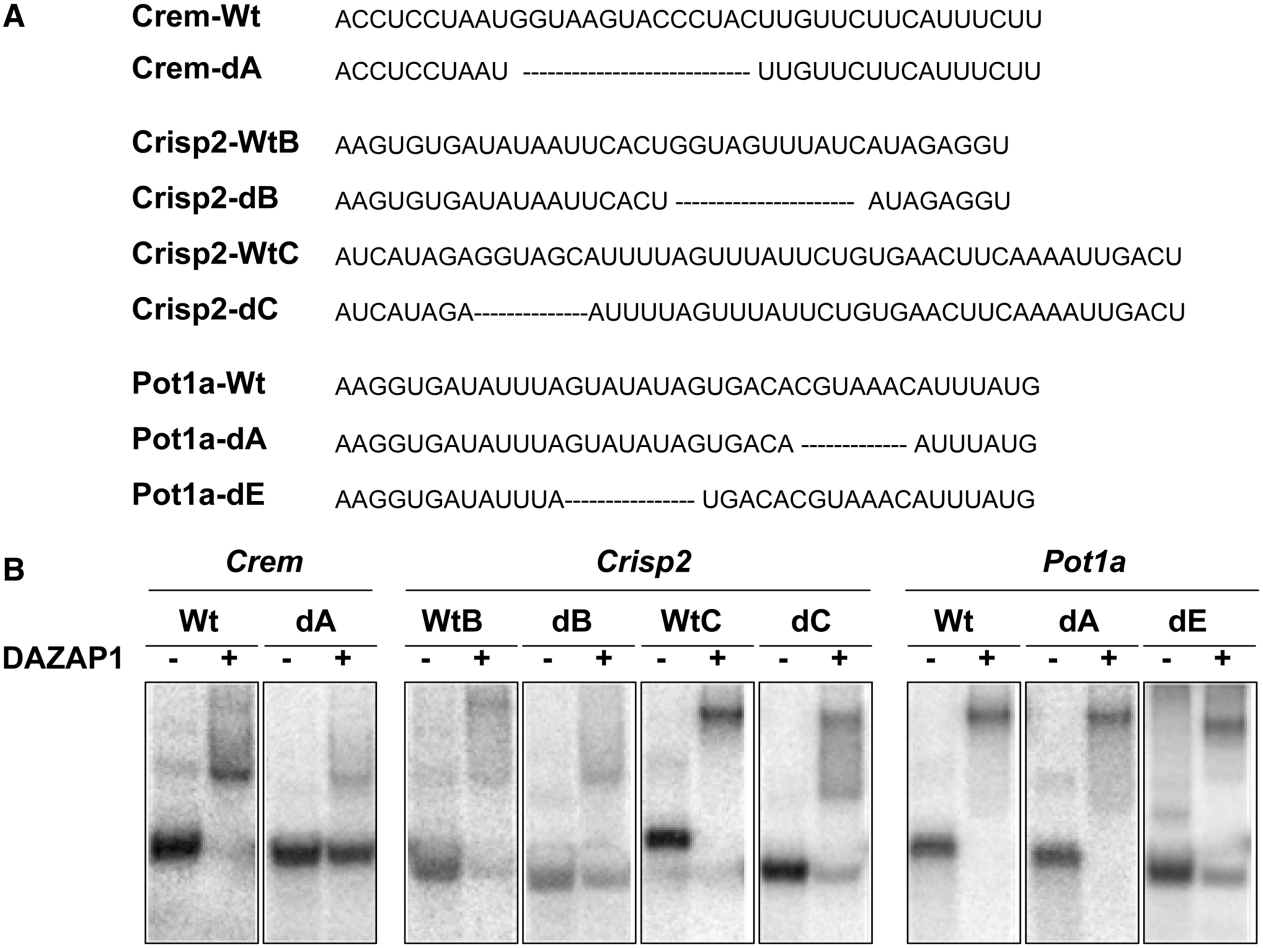
EMSA assays on DAZAP1-RNA binding. (**A**) Sequences of the RNA probes from the three DAZAP1 target genes. Deleted regions are indicated with doted lines. (**B**) EMSA gel patterns. ^32^P-labelled probes were incubated with buffer only (−) or 500 nM GST-DAZAP1 (+) and analysed by 5% native PAGE. Determination of the dissociation constants of the binding between DAZAP1 and the various RNA probes is shown in Supplementary Figure S4.

For *Crem*, P2 from intron 3 and P5 from intron 4 bound most strongly to DAZAP1 (Figure 4A, left panel). We constructed four minigenes with small deletions, including segment A and segment B in intron 3 and segment C and segment D in intron 4, which contained putative DAZAP1-binding sequences. Deletion of segment A, but not segment B, C or D, in the minigene significantly increased exon 4 inclusion from 59.1 ± 4.1% to 86.0 ± 0.3% (*P* = 0.0068) in the absence of exogenous DAZAP1 (Figure 4A, middle panel), suggesting that segment A contained an ISS. Deletion of segment A but not segment B, C or D also significantly reduced the exon 4 inclusion promoted by the exogenous DAZAP1, with the relative change of PSI decreased from 1.65 ± 0.14 to 1.11 ± 0.01, *P* = 0.018 (Figure 4A, right panel). To demonstrate direct binding between segment A and DAZAP1, we performed EMSA assays using short probes (Crem-Wt and Crem-dA, Figure 5A) to minimize possible presence of additional DAZAP1 binding sites. As shown in Figure 5B and Supplementary Figure S4, Crem-Wt bound strongly with DAZAP1 with a dissociation constant (Kd) of 18.93 ± 3.51 nM, whereas Crem-dA lacking segment A showed much weaker binding (Kd = 168.8 ± 40.4 nM, *P* = 0.0221). The results suggest that DAZAP1 binds to segment A in *Crem* intron 3 to mediate exon 4 inclusion.

For *Crisp2*, DAZAP1 bound most strongly to P1 from intron 8 and P5 from intron 9 (Figure 4B, left panel). Deletion of segment B or segment C in intron 9, but not segment A in intron 8, significantly increased exon 9 inclusion in the absence of exogenous DAZAP1 from 20 ± 1.4% to 33.4 ± 0.6% (*P* = 0.0038) and 35.2 ± 1.5% (*P* = 0.0069), respectively, suggesting the presence of splicing silencers in both segments B and C (Figure 4B, middle panel). Deletion of either segment B or segment C also significantly reduced the DAZAP1 promoted exon 9 inclusion, with the relative change of PSI decreased from 2.46 ± 0.18 to 1.46 ± 0.06 (*P* = 0.0072) and 1.50 ± 0.13 (*P* = 0.0072), respectively (Figure 4B, right panel). EMSA assays showed that DAZAP1 bound to both Crisp2-WtB containing segment B (Kd = 33.68 ± 8.95 nM) and Crisp2-WtC containing segment C (Kd = 15.05 ± 0.66 nM) (Figure 5 and Supplementary Figure S4). The presence of multiple major shifted bands hinted at the existence of more than one DAZAP1 binding site in both RNA probes. Nonetheless, deletion of segment B in Crisp2-dB (Kd = 81.82 ± 8.43 nM, *P* = 0.0401) and segment C in Crisp2-dC (Kd = 70.30 ± 8.25 nM, *P* = 0.0064) significantly reduced DAZAP1 binding affinities, supporting the binding of DAZAP1 to both segment B and segment C. Together with the splicing assays, the results suggest that DAZAP1 binds to segments B and C to mediate the splicing of exon 9.

For *Pot1a*, P3 and P4 from intron 4 bound most strongly to DAZAP1 (Figure 4C, left panel). Deletion of segment B, C or D had little effects on minigene exon 4 inclusion (Figure 4C, middle panel). Deletion of segment A caused a modest increase in exon 4 inclusion in the absence of exogenous DAZAP1 from 58.5 ± 0.4% to 64.3 ± 0.7%, *P* = 0.043, and reduced DAZAP1 promoted exon 4 inclusion from 1.40 ± 0.003 to 1.24 ± 0.03, *P* = 0.022 (Figure 4C, right panel). However, additional EMSA assays showed similar binding of DAZAP1 to Pot1a-Wt with segment A (Kd = 7.82 ± 2.02 nM) and Pot1a-dA without segment A (Kd = 7.04 ± 1.01 nM) (Figure 5 and Supplementary Figure S4). On the other hand, deletion of segment E dramatically increased exon 4 inclusion in the absence of exogenous DAZAP1 from 58.5 ± 0.4% to 94.2 ± 0.1%, *P* < 0.0001, indicating the presence of a strong ISS (Figure 4C, middle panel). Addition of exogenous DAZAP1 had little effects on exon 4 inclusion, with DAZAP1 promoted exon 4 inclusion being significantly reduced from 1.40 ± 0.003 to 1.03 ± 0.003 (*P* = 0.0004). Deletion of segment E in Pot1a-dE also significantly decreased DAZAP1 binding affinity, with the Kd increased from 7.82 ± 2.02 nM to 301.2 ± 74.4 nM (*P* = 0.0200) (Figure 5 and Supplementary Figure S4). The results suggest that DAZAP1 modulates exon 4 inclusion through its binding to segment E.

In summary, our EMSA and minigene deletion analyses identified a segment upstream of *Crem* exon 4, two segments downstream of *Crisp2* exon 9 and one segment downstream of *Pot1a* exon 4 that DAZAP1 bound to mediate exon inclusion. These segments had different DNA sequences and all appeared to function as ISSs.

## DISCUSSION

We searched for the natural substrates of DAZAP1 using microarray approaches and identified only three genes of which the splicing is regulated by DAZAP1. These genes likely represent only a small fraction of genes regulated by DAZAP1. In our initial GeneChip microarray screening, we found that >2000 genes had >2-fold changes in expression level in the *Dazap1* mutant testes. The changes in the expression of some, if not most, of the genes could be due to secondary effect of DAZAP1 deficiency, and the underlying mechanisms remain largely unknown. Of the 13 genes selected for further study, we did not observe significant differences in their transcription rates or nuclear export, and identified aberrant splicing in only one of them. Our search for DAZAP1 targets with the exon microarray was not very productive either. Of the 12 genes showing the highest differences in the inclusion of one of their exons, we were able to confirm only two of them. The failure in some instances could be attributed to the low presentation of the differentially expressed exons in the total transcripts. We noted that of the >2000 differentially regulated exons detected by the exon array, close to 20% of them are alternative first exons. This raises the possibility that DAZAP1 may be involved in RNA transcription and promoter selection. A role of DAZAP1 in transcription is also supported by the exclusion of DAZAP1 from the transcriptionally inactive XY bodies in pachytene spermatocytes (12) and the fusion of *DAZAP1* to the *MEF2D* oncogene in a pre-B acute lymphoblastic leukaemia (14,15), and warrants further investigation. Our search for DAZAP1 splicing targets is by no mean thorough. We have performed exon arrays only on testis RNAs and would miss genes regulated by DAZAP1 in other tissues. In addition, the Affymetrix mouse exon array contains only probes that have been identified in cDNA clones or ESTs and will not detect cryptic exons that are included in the transcripts due to DAZAP1 deficiency. Future deep sequencing of RNAs isolated from different tissues of *Wt* and *Dazap1* mutant mice shall provide a more comprehensive list of DAZAP1’s target genes.

While the manuscript was in preparation, Wang et al. reported their identification of six putative DAZAP1 splicing targets in humans using a different approach (43). The group have designed an ingenuous fluorescence-activated screen to identify ISEs in HEK-293T cells and found that the core motif sequence GTAACG in one of the identified ISEs bound DAZAP1, RBM4 and hnRNPD0. They subsequently identified six human genes, including *CORO6*, *SF1*, *ANKS3*, *ZBTB17*, *LAMP1* and *NOL8*, that contained the GTAACG sequence near or within one of their exons and showed that overexpression of DAZAP1 in HeLa cells enhanced the inclusion of the respective exons in the transcripts of the endogenous genes. We thus tested whether DAZAP1 also regulated the splicing of the mouse homologues of these human genes. None of these genes were found to be differentially expressed at the exon level in our exon array analyses. In addition, we observed similar splicing patterns of these genes in the testes of *Dazap1 Wt* and *Fn* mutant mice and the MEFs derived from *Dazap1 Wt* and *null* mutant mice (Supplementary Figure S5). Our failure to detect obvious impact of DAZAP1 on the splicing of the mouse homologues of the six putative DAZAP1 targets in humans suggests species or tissue/cell differences in DAZAP1 targets.

So far there have been five studies, including this one, on the effect of DAZAP1 on RNA splicing. The first two studies found negative effects whereas the remaining three observed positive effects. The negative cases involve the *NF1* exon 37 and *BRCA1* exon 18 mutations that created new binding sites for DAZAP1 and hnRNPA1/A2 and caused skipping of the respective exons (17,18). *In vitro* studies on splicing minigenes in HeLa cells showed that knocking down DAZAP1 alone had little effects on the inclusion of *NF1* and *BRCA1* exons, and only when DAZAP1 was knocked down together with hnRNPA1 and hnRNP A2 did one see an increase in exon inclusion. On the other hand, overexpression of DAZAP1 alone suppressed the inclusion of *NF1* exon 37 as well as its neighbouring exon 36, supporting a negative effect of DAZAP1 on exon inclusion. The third case involves the Alu-derived ISE in *ATM* that caused the inclusion of a cryptic exon (19). Overexpression of hnRNPA1 suppressed the inclusion of the cryptic exon in HeLa cells whereas overexpression of DAZAP1 had no effect on the splicing. On the other hand, knocking down hnRNPA1 and DAZAP1 significantly enhanced and decreased, respectively, the inclusion of the cryptic exon, indicating opposite effect of the two proteins on exon inclusion. The recent study by Wang et al. (43) mentioned above as well as our study also observed a positive effect of DAZAP1 on exon inclusion.

We noticed that the impact of DAZAP1 on exon inclusion differed for different genes and different cells. For the endogenous transcripts in mouse testes, DAZAP1 deficiency in the mutant mice caused exon skipping in a minor fraction of *Crem* transcripts but major fractions of *Crisp2* and *Pot1a* transcripts (Figure 1C). For the expression of splicing reporter minigenes in COS7 cells without exogenous DAZAP1, different genes showed different PSIs, with the targeted exons included in most of the *Pot1a* transcripts, about half of the *Crem* transcripts, and a small fraction of the *Crisp2* transcripts, and overexpression of DAZAP1 significantly enhanced exon inclusion in the *Crem* and *Crisp2* minigenes (Figure 3B and Supplementary Figure S3A). However, the absence of DAZAP1 in MEFs only affected the exon inclusion in the *Pot1a* minigene, with the *Crem* and *Crisp2* minigenes showing similar splicing patterns in *Wt* and *Null* MEFs (Figure 3C and Supplementary Figure S3B). Although DAZAP1 bound to multiple regions around each of the targeted exons, results of minigene deletion analyses suggested that only one or two intronic segments were involved in modulating exon inclusion. It is interesting that all these segments appear to contain splicing silencers. The effect of DAZAP1 on exon inclusion has been suggested to be modulated by other splicing factors present in the cells/tissues, such as hnRNPA1 and RBM4 (43). hnRNPA1 is a well-studied splicing factor that negatively regulates splicing by binding to ESSs and ISSs, and some of its binding sequences are also recognized by DAZAP1 (17–19). It is possible that DAZAP1 enhances exon inclusion of some of its target genes by competing with hnRNAP1 for the binding to ISSs. DAZAP1 has also been found to compete with RBM4 for the same ISE and antagonize each other on exon inclusion (43). Whether hnRNPA1, RMB4, or other splicing factors are involved in the splicing of the three exons studied here requires further investigation.

As mentioned above, most *Dazap1* deficient mice die soon after birth, and the few survivors inevitably manifest grow retardation and spermatogenic arrest right before meiosis I (20). Of the three DAZAP1 target genes identified here, anomalous splicing of *Crem* and *Crisp2* unlikely contribute to the spermatogenic phenotype of the *Dazap1* mutant mice because both genes are mainly expressed in postmeiotic germ cells. On the other hand, abnormalities in *Pot1a* splicing likely play a major role in both perinatal lethality and growth retardation of the mutants. POT1A contains two N-terminal oligonucleotide/oligosaccharide binding folds (OB-folds) that are essential for telomere binding. The *Pot1a* gene (NM_133931) contains eight ATG start codons in the 5′ UTR region that are followed by nearby in-frame stop codons; thus, its translation initiates at the ninth ATG located in exon 4 (40,41). Exon 4 skipping does not put any of the 5′ UTR ATGs in-frame with the open reading frame. Therefore, the translation of the *Pot1a* transcript without exon 4 is expected to initiate at an in-frame ATG in exon 6, producing a protein without the first 59 amino acid residues. It has been shown previously that POT1A truncated for the N-terminal 48 residues, including a portion of the first OB fold, failed to bind single-stranded telomeric DNA (40). Thus, the protein product of the *Pot1a* transcript lacking exon 4 is likely to be non-functional even if it were generated and stably present in the cells. *Dazap1 Fn* mutants still produce a low level of the full-length *Pot1a* transcript and protein, which may be sufficient to prevent the embryonic lethality phenotype observed in *Pot1a* KO mice. Whether POT1A deficiency also contributes to the spermatogenic phenotype of *Fn* mice remains to be determined. The human *POT1* gene is expressed most abundantly in the testes (41), whereas the level of *Pot1a* transcript in mouse testes is comparable with those in other tissues (40). The role for POT1A during spermatogenesis awaits further investigation.

## SUPPLEMENTARY DATA

Supplementary Data are available at NAR Online.

## FUNDING

National Science Council [96-2311-B-011-026-MY3]; Academia Sinica in Taiwan. Funding for open access charge: Academia Sinica.

*Conflict of interest statement*. None declared.

## Supplementary Material

Supplementary Data
