# Supplementary material for: DAZAP1 regulates the splicing of Crem, Crisp2 and Pot1a transcripts
Source: Nucleic Acids Res. 2013 Aug 21;41(21):9858–69. doi: 10.1093/nar/gkt746 (PMC3834821; doi:10.1093/nar/gkt746)
Supplement: Supplementary Data [file supp_41_21_9858__index.html]

DAZAP1 regulates the splicing of Crem, Crisp2 and Pot1a transcripts — DAZAP1 regulates the splicing of Crem, Crisp2 and Pot1a transcripts — Supplementary Data 

# DAZAP1 regulates the splicing of *Crem*, *Crisp2* and *Pot1a* transcripts

## Supplementary Data

files

**Files in this Data Supplement:**

- Supplementary Data - pdf file
