# Supplementary material for: DAZAP1 regulates the splicing of Crem, Crisp2 and Pot1a transcripts
Source: Nucleic Acids Res. 2013 Aug 21;41(21):9858–69. doi: 10.1093/nar/gkt746 (PMC3834821; doi:10.1093/nar/gkt746)
Supplement: Supplementary Data [file supp_gkt746_nar-02056-a-2013-File007.pdf]

Supplementary Data (Chen, Yu & Yen)

Supplementary Table S1: RT-PCR primers for specific mouse genes

Gene	Primer ID	Sequence (5'-3')	Starting Location (direction)
Crem	PrCrem-2	GGA GCT CGG ATC TGG TAA G	Exon 9 (R)
	PrCrem-3	CAA CCG CAT CAG AGC TGA C	Exon 1 (F)
	PrCremE3F	ACG CTA GCG CAA ATG ACC ATG GAA AC	Exon 3 (F)
	PrCremE5R	ACG GGC CCT CTT CGT GAA AGA ATT TC	Exon 5 (R)
Crisp2	PrCrisp2-1	TCA GCT GTC GGA CAT TAC AC	Exon 7 (F)
	PrCrisp2-2	CCC CAT CAG TCT CAA CTA AG	Exon 10 (R)
	PrCrisp2-3	AGG CGG TTA TCG TTG CTC AC	Exon 1 (F)
Pot1a	PrPot1a-1	CAT GCT ACA GAG GCT GAA ACC	Exon 3 (F)
	PrPot1a-2	CCT GTG GAA GCG AAC AAT GTC	Exon 6 (R)
	PrPot1a-3n	CGC CTA GGT CCA GAC TTT AG	Exon 1 (F)
	PrPot1a-4	TTC GTC GCT CTG TTC CGA TG	Exon 18 (R)
Actin	PrbActin-F2	CCA ACC GTG AAA AGA TGA CCC	(F)
	PrbActin-R	CAA TAG TGA TGA CCT GGC CGT	(R)
Zbtb17	PrZbtb17-1	GAT CCT ACA GGC ACA CTG TC	Exon 2 (F)
	PrZbtb17-2	ATC CTG CAT CTG GAG GAA GC	Exon 4 (R)
Lamp1	PrLamp1-1	GAC CCC AGC CTC ACA ATT AC	Exon 3 (F)
	PrLamp1-2	TCC ATC CTG TGT GCA GTG TG	Exon 5 (R)
Sf1	PrSf1-1	GGA ACC AAG ACA CAA TGG AG	Exon 2 (F)
	PrSf1-2	CCC CTC GCT GTT GTA GAT TG	Exon 4 (R)
Anks3	PrAnks3-1	AAG GAC AGA CTC CAC TGA TG	Exon 3 (F)
	PrAnks3-2	CTG CTT CCA TCA AAG GAG TG	Exon 5 (R)
Coro6	PrCoro6-1	ACC ACC TGC AAG GAC AAG AC	Exon 4 (F)
	PrCoro6-2	GGA GTC AGG ATC GTA GAA TG	Exon 6 (R)
Nol8	PrNol8-1	AAT GGA AAG GTG GAA CAC TAC	Exon 4 (F)
	PrNol8-2	AAG AAC AGG TAA GAC TCT TCC	Exon 6 (R)

PCR conditions: 95℃ (5 min) for the initial hot start; 95℃ (20 s), 60℃ (20 s), and 72℃ (30 s-2 min 30 s) for 30 cycles; and 72℃ (10 min) for the final extension.

Supplementary Table S2: PCR primers for the construction[1] and splicing assay[2] of minigenes

Gene	Primer ID	Restriction site	Sequence (5'-3')[3]
Crem	PrCremE3F*	NheI	ACg cta gcG CAA ATG ACC ATG GAA AC
	PrCremE3R	KpnI	ATg gta ccA GCT GTG AAC CGT GAA AG
	PrCremE4F	KpnI	ACg gta ccA GAT AGA AAC TCC CAC TG
	PrCremE4R	XhoI	ATc tcg agC CAG CCA GGT AAA GTT TC
	PrCremE5F	XhoI	CGc tcg agT ATT GGA AGG CAA CAT TG
	PrCremE5R	ApaI	ACg ggc ccT CTT CGT GAA AGA ATT TC
Crisp2	PrCrisp2-E8F*	NheI	GCC gct agc CTT GTT TGG TAT TCA TC
	PrCrisp2-E8R	XhoI	ATc tcg agG GTC CAC CAT CAA ACT TG
	PrCrisp2-E910F2	XhoI	ctc gag AAC ACA GGT GGA GGG AAC
	PrCrisp2-E910R	ApaI	AAT ggg ccc TGT GGT AGC TTG AGT TC
Pot1a	PrPot1a-E34F*	NheI	gct agc AGG CTG GAT TGT CCT AAC
	PrPot1a-E34R	XhoI	ctc gag CAC TAC TGA GTA CCC AAC
	PrPot1a-I4F2	XhoI	CCG ctc gag ATT TAA CAA ATT GTA TAA ATA AAC
	PrPot1a-I4R2	XhoI	CGc tcg agT ATT TTA ATC AGT CTT TAA TAT AC
	PrPot1a-E5F	XhoI	ctc gag GCA AAG CCC TTC TCT ATC TAC
	PrPot1a-E5R	ApaI	ggg ccc CAG TTC CTT TGC TAA CAT ACG

1. For minigene construction, PCRs were performed under the following conditions: 95℃ (5 min) for the initial hot start; 95℃ (20 s), 60℃ (20 s), and 72℃ (3 min) for 35 cycles; and 72℃ (10 min) for the final extension.
2. For splicing assays, PCRs were performed with the forward primers marked with an asterisk and a reverse primer BGH-R (5'-TAGAAGGCACAGTCGAGG) containing the vector sequence downstream of the multiple cloning sites. The PCR conditions consist of 95℃ (5 min) for the initial hot start; 95℃ (20 s), 60℃ (20 s), and 72℃ (1 min) for 25 (*Crisp2 and Pot1a*) or 34 *(Crem*) cycles; and 72℃ (10 min) for the final extension.
3. The restriction sites are shown in lower cases.

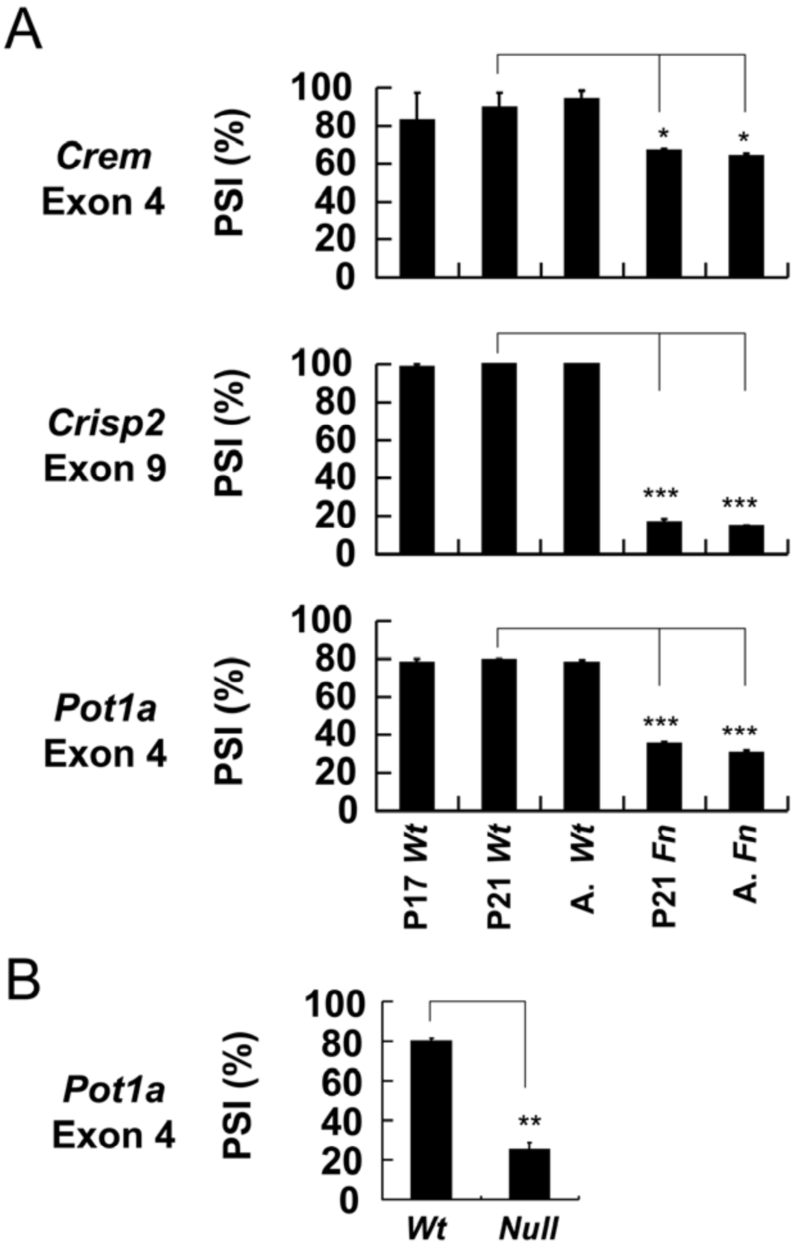

Supplementary Figure S1. Percentage of Splicing Inclusion (PSI) of DAZAP1 target exons. (A) *Crem*, *Crisp2*, and *Pot1a* transcripts in the testes of wild-type (*Wt*) and *Fn* mutant mice at various ages were RT-PCR amplified across the indicated exons as shown in Figure 1C, the fragments with and without the indicated exon were quantified to determine the PSIs. (B) *Pot1a* transcripts in MEFs derived from *Wt* and *Null* embryos were RT-PCR amplified across exon 4 to determine PSIs of exon 4. The results shown represent the average of three independent experiments. Statistical significance of the difference was determined using the paired t test. * denotes $p < 0.05$ and *** denotes $p < 0.001$.

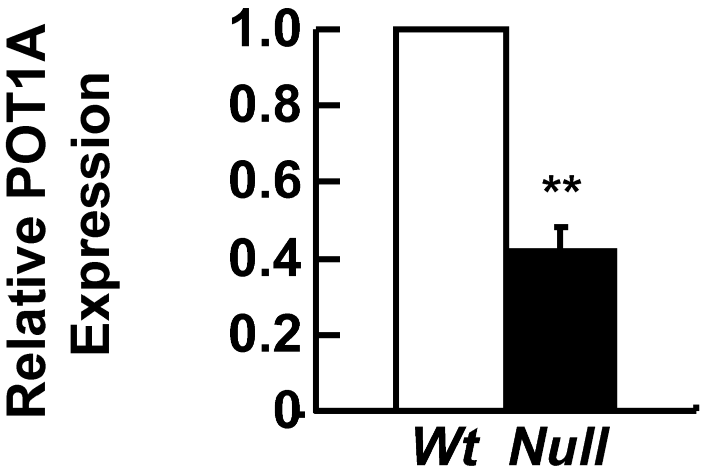

Supplementary Figure S2. Quantification of the relative levels of POT1A in MEFs derived from wild-type and *Dazap1 null* mutant embryos, as detected by western blotting shown in Fig. 2 in the main manuscript. The level of POT1A in the *Wt* MEF was used as the reference (1.0). The results shown represent the average of three independent experiments. Statistical significance of the difference was determined using the paired t test. ** denotes p=0.0039.

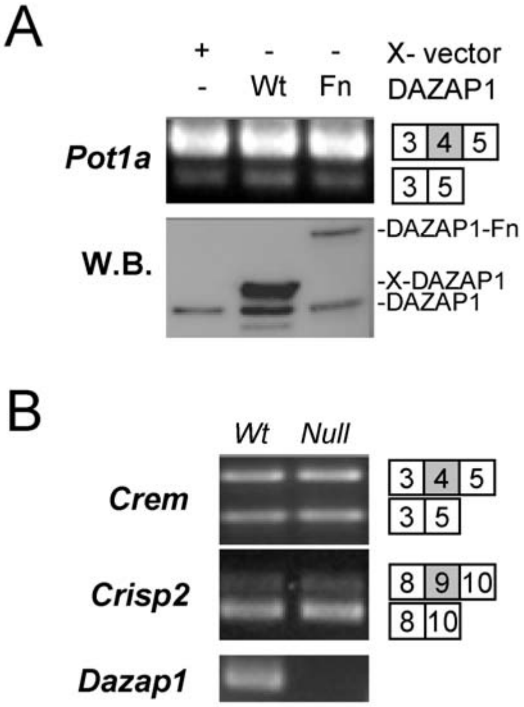

Supplementary Figure S3. Minigene splicing assay of DAZAP1 target exons. (A) COS7 cells were transfected with a *Pot1a* minigene together with an empty Xpress cloning vector (X-vector) or the expression vector for Xpress-tagged wild-type DAZAP1 (X-DAZAP1) or DAZAP1-Fn mutant. Twenty-four hours after transfection, the cells were harvested and the expression of the minigene was detected by RT-PCR. The endogenous and exogenous DAZAP1 proteins were detected by western blotting (W.B.) using an anti-DAZAP1 antibody. (B) MEF cells derived from *Dazap1 Wt* and *null* mutant mice were transfected with *Crem* or *Crisp2* minigene and the expression of the minigenes was detected by RT-PCR. Additional RT-PCR shows the expression of *Dazap1* transcripts in *Wt* but not *null* MEFs.

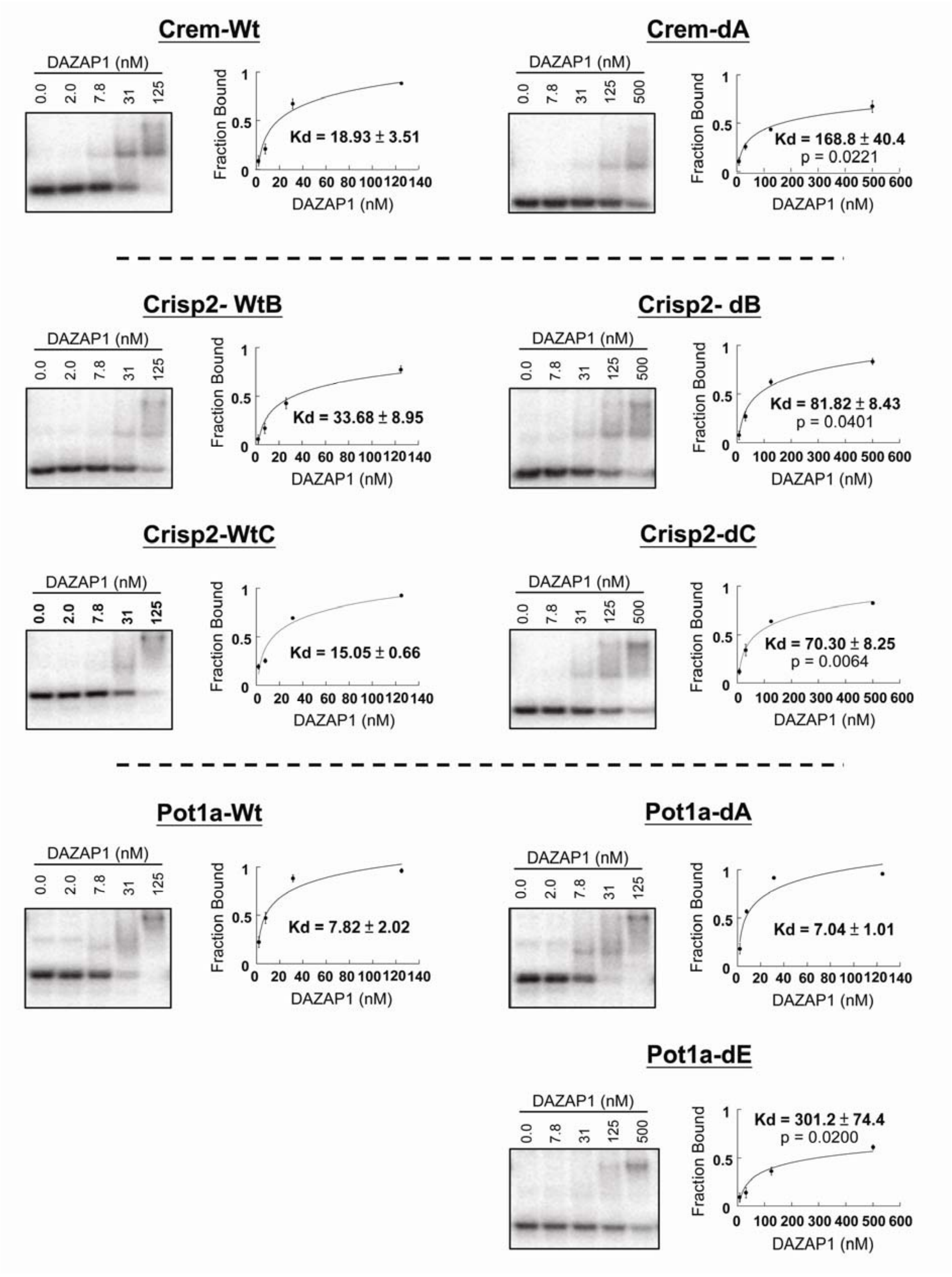

Supplementary Figure S4. Determination of the dissociation constants (Kd) of DAZAP1-RNA binding. ^{32}P-labeled RNA probes were incubated with various concentrations of GST-DAZAP1 and the mixtures were subjected to EMSA analysis. For each panel, the EMSA pattern is shown on left and

quantification of the bound fraction is shown on right. Kd, the protein concentration at which 50% of the RNA is bound, is indicated. The results shown represent the average of three independent experiments. Statistical significance of the difference between the Kds of the probes without and with the deletion was determined using the paired t test.

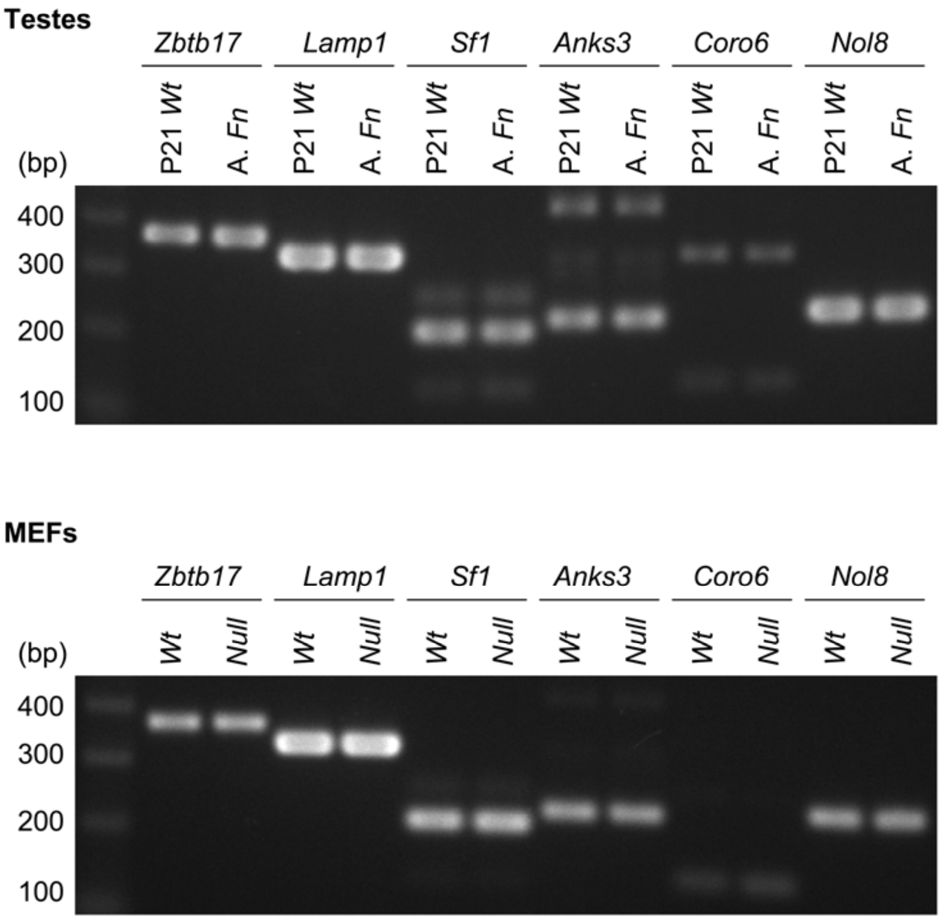

Supplementary Figure S5. RT-PCR analyses of the expression of various genes in the testes of P21 *Wt* mice and adult *Dazap1 Fn* mice (top panel), and MEFs derived from *Dazap1 Wt* and *null* mice (bottom panel). Sequences of the primers and PCR conditions are given in Supplementary Table S1.
